# Evaluation of a new semi-automatic strategy for quantitative measurement of infarct size in patients with acute and chronic myocardial infarction using cardiac magnetic resonance imaging

**DOI:** 10.1186/1532-429X-15-S1-P201

**Published:** 2013-01-30

**Authors:** Gunnar Lund, Dennis Saering, Kai Muellerleile, Julia Cuerlis, Dominik Barz, Peter Bannas, Ulf K Radunski, Karsten Sydow, Gerhard Adam

**Affiliations:** 1 Radiology, University Hospital, Hamburg, Germany; 2 Clinical Neuroscience, University Hospital, Hamburg, Germany; 3 Cardiology, University Hospital, Hamburg, Germany

## Background

Cardiac magnetic resonance imaging (CMR) with delayed gadolinium enhancement (DE) enables accurate infarct size measurement, however, the evaluation of infarct size using a threshold method is time consuming and relies on accurate placement of normal regions of interest and manual delineation of the infarct size.

The purpose of the study was to analyze the accuracy and expenditure of time of a new evaluation strategy for infarct size measurement using delayed enhancement CMR in comparison to a standard evaluation.

## Methods

DE-CMR studies from 10 patients with acute myocardial infarction and 10 patients with chronic infarction were quantitatively evaluated by 1 experienced and 2 novel, but trained observers in respect to infarct size using a threshold method. For standard evaluation the signal intensities of remote myocardium were measured in five regions in non-infarcted myocardium. Images were thresholded to a level >2 SDs than that of normal myocardium and the infarct was manually traced (see Figure). The new evaluation algorithm simply required liberal encircling of the infarcted and remote normal myocardium (see Figure). Subsequently, images were automatically thresholded and the infarct area was calculated according to a threshold of >2 SDs. Infarct size, agreement between observers and evaluation time was compared between both evaluation strategies using t-test and Bland-Altman analysis.

**Figure 1:**
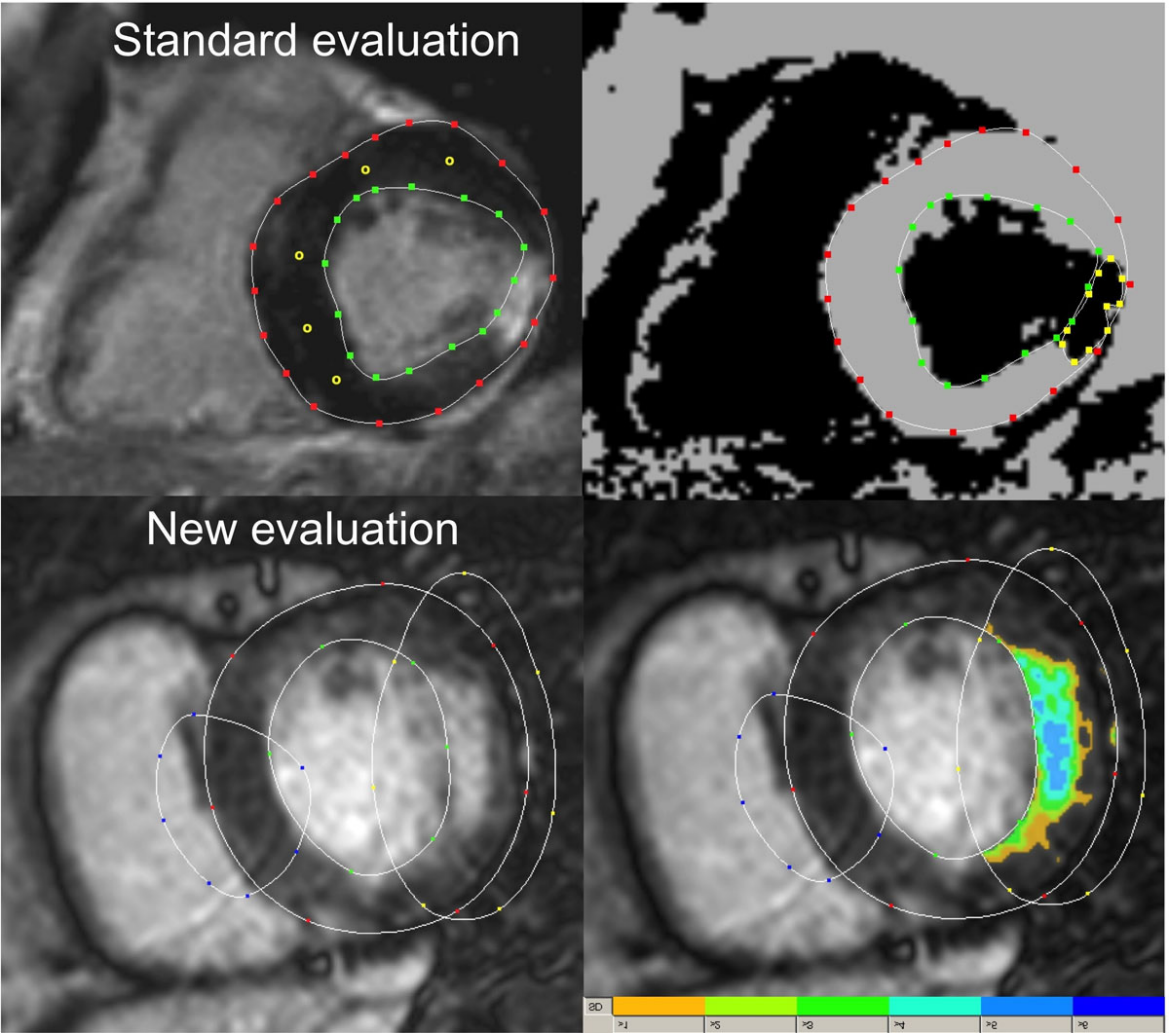
Shows on the upper row the standard evaluation with placement of 5 regions of interest (yellow circles) in remote normal myocardium and manual tracing of the infarct on the threshold image. In the lower row the new evaluation is shown with liberal encircling of the infarcted and remote normal myocardium. The right image shows the automatically thresholded image with color coding of infarct according to the threshold.

## Results

Mean acute infarct size was 19,3 %LV using standard evaluation and 18,0 %LV for the new evaluation (P=ns). In chronic infarction, mean infarct size was 8,6 ± 10,9 %LV for the standard evaluation and 7,8 ±10,0 %LV for the new evaluation (P=ns). Agreement between the experienced and the novel observers was good with -3,0 ± 13,7% for standard evaluation and +3,0 ± 14,1 % for the new evaluation (P=ns). Evaluation time for infarct size measurement was significantly reduced from 15,3 ± 5,5 min to 9,6 ±2,7 min (P<0.001).

## Conclusions

The new semi-automatic evaluation strategy results in similar infarct size in acute and chronic infarction in comparison to standard evaluation. Furthermore, the agreement between observers is very good for the new strategy. The new evaluation strategy is very valuable because the evaluation time is significantly reduced.

## Funding

none

